# Humanoid robots for psychological assessment in mild cognitive impairment: from evaluation to the future of AI-driven data prediction systems

**DOI:** 10.3389/fpsyg.2025.1579626

**Published:** 2025-08-19

**Authors:** Caterina Formica, Fabio Mauro Giambò, Desiree Latella, Lilla Bonanno, Marco Lombardo, Orazio Tomarchio, Angela Marra, Antonella Alagna, Carmen Bonanno, Angelo Quartarone, Silvia Marino

**Affiliations:** ^1^IRCCS Centro Neurolesi “Bonino Pulejo”, Messina, Italy; ^2^Behavior Labs srl, Catania, Italy; ^3^Department of Electrical Engineering, Electronics and Computer Science, University of Catania, Catania, Italy

**Keywords:** artificial intelligence, humanoid robot, mild cognitive impairment, neuropsychological assessment, rehabilitation

## Abstract

**Introduction:**

Neurodegenerative diseases, such as Alzheimer’s Disease (AD), are increasingly prevalent, emphasizing the need for early diagnosis and effective intervention. This study explores the feasibility of using the humanoid robot *Pepper* to administer cognitive assessments for Mild Cognitive Impairment (MCI). Specifically, it evaluates the usability, accuracy, and patient experience of robot-administered cognitive testing compared to traditional assessments conducted by neuropsychologists.

**Methods:**

A total of 100 MCI patients were randomly assigned to two groups: one undergoing the Mini-Mental State Examination (MMSE) with *Pepper* and the other receiving the same test administered by a neuropsychologist. After that participants were submitted to a Satisfaction Questionnaire (SQ) designed to assess their emotional and experiential response to the testing procedure, whether administered by a human or a robot.

**Results:**

The intergroup analysis (EG vs. CG) reveals significant differences in age (*p* = 0.003) and Total SQ (*p* = 0.01), and in SQ2 (*χ*^2^ = 9.76; df = 1; *p* = 0.002), SQ4 (*χ*^2^ = 5.02; df = 1; *p* = 0.02), SQ5 (*χ*^2^ = 25.35; df = 1; *p* < 0.001), SQ6 (*χ*^2^ = 7.68; df = 1; *p* = 0.006) and SQ7 (*χ*^2^ = 7.56; df = 1; *p* = 0.006). Results indicate no significant differences in MMSE scores between the two groups, suggesting comparable cognitive evaluation accuracy. However, participants assessed by *Pepper* reported lower frustration levels and higher satisfaction (90% vs. 40%) compared to those tested by a neuropsychologist. Additionally, 92% of patients in the robot-assisted group expressed willingness to retake the test in the same manner, indicating high acceptability and engagement.

**Discussion:**

These findings suggest that robot-assisted cognitive assessments may enhance patient comfort and accessibility to neuropsychological testing. The integration of Artificial Intelligence (AI) further supports diagnostic accuracy and predictive potential, offering promising avenues for early intervention in neurodegenerative diseases.

## 1 Introduction

Recently, the number of people with cognitive decline who develop dementia has increased, considering the age of the global population. Neurodegenerative disorders have been defined by the World Health Organization (WHO) as a global public health priority (WHO, 2020). An epidemiological study in Italy estimated that, by 2051, there will be 280 elderly people per 100 young people with an increase in chronic and degenerative diseases, including dementia (Gervasi et al., 2020). The number of patients with neurodegenerative disorders, such as Alzheimer’s disease and Parkinson’s disease, is estimated to be over 1 million between Alzheimer Disease (AD) and Parkinson Disease (PD) (Fania et al., 2023). The presence of majority of aging population has a significant impact in healthcare systems in terms of economics, pharmacotherapy and social burden. All these considerations have caused the National Health System (NHS) to increase the accuracy and earliness of diagnosis, in response to the increase in patients with dementia. Diagnosis is based on the collection of clinical comorbidity data, neuropsychological assessments, and neuroimaging analyses. Evidence suggested that mild cognitive impairment (MCI) represents an early stage of AD (Lopez, 2013; Morris et al., 2001). The detection of MCI may facilitate the identification and classification of people at a higher risk of developing dementia (Berres et al., 2021; Yang et al., 2020). From the perspective of differential and early diagnosis, MCI shows typical cognitive markers, such as deficits in episodic memory, slow thinking, and occasionally language deficits, but does not interfere with daily living activities (Ataollahi Eshkoor et al., 2015). These cognitive and behavioral hallmarks are typically detected through first-level neuropsychological screening, usually performed in clinical settings using standardized paper-and-pencil tests. Nevertheless, these preliminary evaluations are essential to identify individuals at risk of neurodegenerative disorders and to guide timely referral to second-level outpatient of Neurodegenerative Disorders, where patients undergo a comprehensive diagnostic workup and individualized care planning. Recent studies have highlighted significant cross-national differences in the attitudes of both older adults and healthcare professionals toward neuropsychological assessments and the use of assistive technologies. For example, in Northern European countries and the United States, cognitive screening and functional assessments (e.g., MMSE, MoCA, IADL scales) are more routinely implemented in clinical practice, whereas in some Southern and Eastern European countries, their use is less standardized or accepted (Katsanos et al., 2023). The process of cognitive assessment, especially for elderly patients with MCI, often requires significant time and effort, with many patients accompanied by caregivers due to the complexity of the testing procedures and the potential cognitive and emotional burden involved. The need to create a smart and more readily available method in the early stages of dementia is growing. Moreover, older adults’ acceptance of telemedicine, digital therapeutics depend on cultural factors, prior exposure, and healthcare infrastructure. Some studies have investigated the effectiveness of using devices, tablets (Koo and Vizer, 2019), smartphones (Formica et al., 2024), and virtual reality tools (Chua et al., 2019; Tarnanas et al., 2013) to assess and monitor cognitive functions over time, rather than paper-pencil tests. However, many of these methods are unsuitable for daily use and do not significantly reduce healthcare costs, because the use of these technologies requires specific skills (not easy for older people). These challenges often require assistance from an operator during cognitive assessments, as many older patients face difficulties in independently interacting with electronic devices. This dependency can lead to frustration, impacting the patient’s performance and potentially invalidating the test results. In contrast, humanoid robots offer a solution by providing a more intuitive and automated testing experience, thus reducing reliance on operators and minimizing sources of frustration. Given the emotionally sensitive nature of cognitive assessments, the perception of reduced social judgment during human–robot interaction may enhance patient comfort; offering an advantage in the context of neuropsychological screening for cognitive decline (Varrasi et al., 2018). Based on this perspective, robotic technology can provide the administration of cognitive tests offering an embodied presence to improve engagement during evaluation, making them more interactive and stimulating. In addition, the participants’ responses were immediately recorded and scored by the robot for further direct specialist analysis (Formica et al., 2024). These methods require less technological competence than interacting with other electronic devices (computers and tablet virtual reality systems) (Coronado et al., 2022). Robotic technology AI-driven, offers an innovative solution by allowing for the automatic administration of cognitive tests, real-time scoring, and immediate feedback on the results. Additionally, the robot’s ability to track long-term cognitive performance and generate predictive models using clinical data helps clinicians make more informed decisions. This capability significantly reduces the cognitive load on patients, minimizes potential distractions, and enhances the objectivity and reliability of the cognitive assessments, offering a clear improvement over traditional methods that require human assistance (Giannouli, 2023; Giannouli and Kampakis, 2024). This approach could be useful for the earlier detection of cognitive impairment, potentially predicting the process of cognitive decline more accurately (Formica et al., 2023). Furthermore, the combination of robotics and AI can facilitate the development of personalized diagnostic tools that can be adapted to the cognitive profile of each patient, offering tailored interventions at an earlier stage (Graham et al., 2020). Such advancements aim to improve diagnostic accuracy as well as have the potential to slow the progression of neurodegenerative diseases by enabling timely and effectively treatment strategies. Studies have used robots for cognitive assessment in healthy older adults to demonstrate the feasibility of their application (Sorrentino et al., 2021; Takaeda et al., 2019), whereas studies on robotic assessments of MCI are limited. These gaps led us to investigate two important questions: First, we examined the validity of the cognitive tests administered by a humanoid robot using the performance of a paper-pencil standardized neuropsychological test as a benchmark. Second, we examined the feasibility and usability of a humanoid robot in the assessment of cognitive impairment in patients with MCI and detected differences between cognitive tests administered by robots and healthcare professionals. Given previous findings suggesting that individuals may feel less judged and more at ease when interacting with social robots (Holthöwer and Van Doorn, 2023; Desideri et al., 2019), we hypothesized that patients with MCI would report higher emotional comfort, motivation, and overall acceptability, and lower perceived judgment and frustration when undergoing the MMSE with a humanoid robot compared to a human examiner. Considering the emotional vulnerability and sensitivity to evaluation often present in MCI, we expected that the robot-mediated condition could offer a more neutral and supportive testing experience.

## 2 Materials and methods

### 2.1 Participants

The sample size was estimated *a priori* using G*Power software (version 3.1.9), considering the primary outcome of MMSE score comparison between the Experimental Group (EG) and Control Group (CG). Assuming a two-tailed independent-samples t-test, an effect size of *d* = 0.6 (medium-to-large), an alpha level of 0.05 and a power of 0.80, the required sample size was 45 participants per group (90 total). We enrolled 100 participants (50 per group) to account for possible dropouts and to ensure sufficient statistical power. Additionally, the final sample size also satisfies power requirements for detecting medium effects (*w* = 0.3) in chi-square tests comparing categorical variables between groups, responses to usability and acceptability items in the administered questionnaire.

We enrolled 100 participants matched for sex and education. All patients were MCI diagnosis based on neurological assessment conducted by a neurologist. and included in this study based on the following criteria: (1) Level of autonomy in Daily Living Activities (ADL) ≥ 4; (2) presence of memory deficits; and (3) Clinical Dementia Rating (CDR) < 3 scoreclusion criteria were as follows: (1) language deficits, (2) psychiatric disorders, (3) visual and severe hearing impairment, and (4) history of brain injury. Patients were recruited from clinic outpatients of general neurology and neurodegenerative disorders; the neurologist conducted the anamnestic collection data and revealed any exclusion criteria to participate to the study. The neurologist evaluated also the presence of depressive and anxious symptoms that could influence the cognitive performance. This stage had a duration of 30 min. The enrolment stage started on January 2024 to March 2025. Patients signed the written informed consent and before conducting the experiment, patients were randomized in two groups, Experimental Group (EG) and Control Group (CG) in which they were submitted to neuropsychological evaluation through Humanoid Robot “Pepper” and a neuropsychologist, respectively. It was used a simple randomization from Random.org.^1^ The present study represents a predefined secondary analysis of the broader TRAIT “Therapeutic Robot and Artificial Intelligence in experimental Therapy” project (T.R.A.I.T.) (ClinicalTrials.gov Identifier: NCT05788133). The TRAIT protocol encompasses multiple sub-studies aimed at exploring various clinical dimensions. This manuscript focuses on a specific subset of objectives and participants, as outlined in the present methods section whose goal is to perform Machine Learning Model (MLM) for cognitive decline. All procedures were conducted in accordance with the general methodology of the registered protocol. The study was conducted in accordance with the Declaration of Helsinki and approved by the Ethics Committee of the IRCCS Centro Neurolesi Bonino Pulejo (protocol code 48/2021, approved on June 30, 2023).

### 2.2 Humanoid-robot

**Figure 1:**
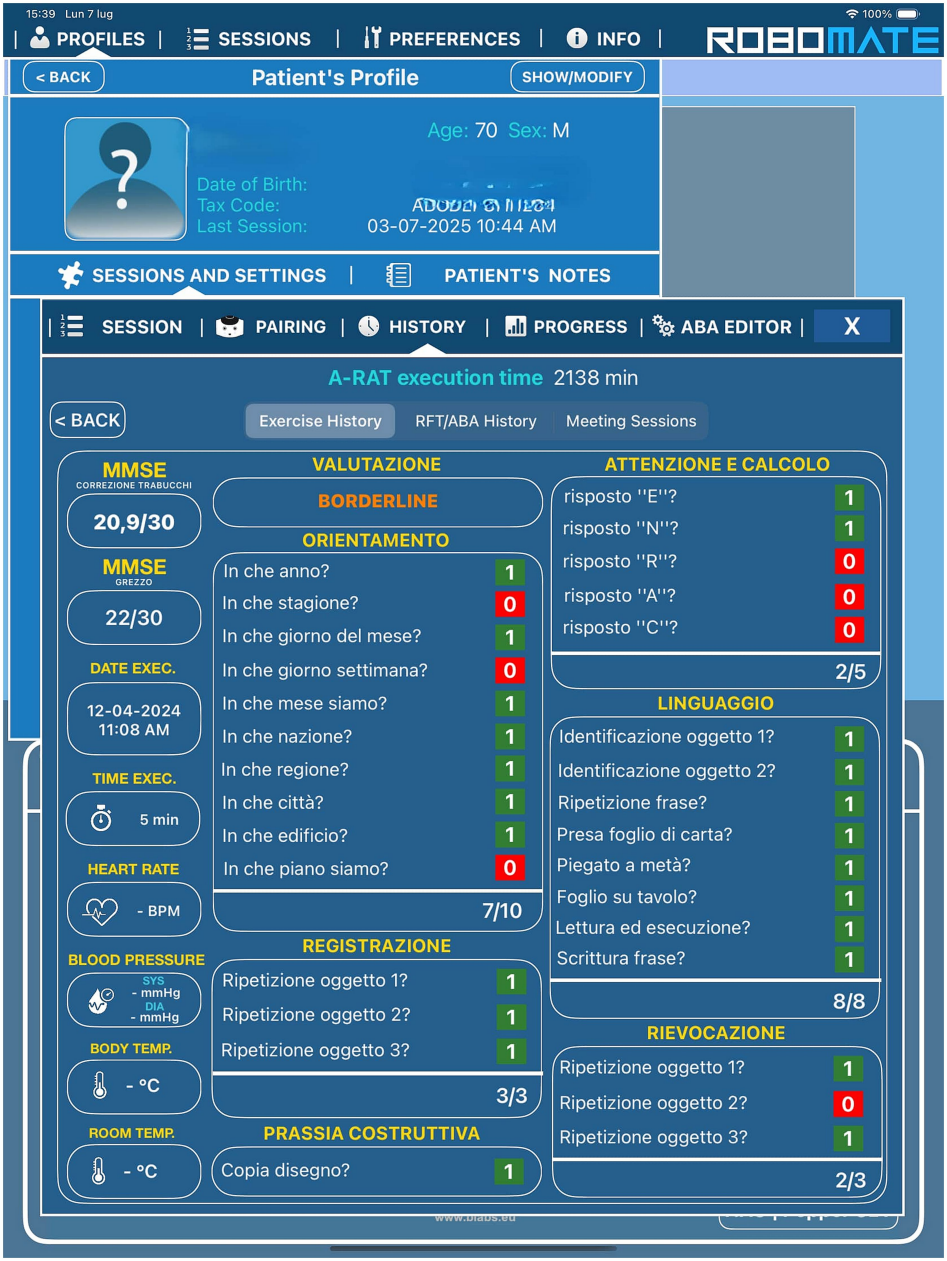
Total and subtest scores of Mini Mental State Examination (MMSE).

Pepper was the first humanoid robot produced by SoftBanks Robotics in Japan in 2014, that is 1.20 m high and weighing 28 kg. This is the first robot that can identify facial expressions and basic human emotions and interact with the environment through machine learning.^2^ This technology enables the analysis of people’s expressions and voice tones using the latest advances and proprietary algorithms in voice and emotion recognition to trigger interactions. The robot is equipped with features and high-level interfaces for multimodal communication with the human surroundings. The touchscreen on his chest displays content that highlights messages and supports speech as a reinforcing and feedback component through which a person can respond and interact during the session. The robot provides feedback through a combination of verbal responses and visual feedback accompanied by head and arm gestures (e.g., nodding, clapping, or performing a victory dance). Pepper is supported by RoboMate software tailored to determine its behavior. It is a Learning Management System (LMS) platform used to obtain real-time predictive results starting from clinical data collection realized through the RoboMate App, which supports the therapist in remotely controlling the movements and voice of the robot, triggering predefined animations, questions, and feedback responses. Specifically, it record patients’ answers and manage their data, including the cognitive performance score collected during the evaluation (Figure 1). The software architecture was robot-centric.

The tests were performed primarily by the robot, and the tablet displayed the data that the robot held in memory. The Choreographe project is created according to Python block logic and timeline blocks, where each block defines one or more input parameters that are processed within the block and provides one or more outputs. The results of each exercise were aggregated according to the objective and stored within a “cloud” service, in a database containing the patient’s biographical data, the rehabilitation objectives defined by the practitioner, and the results of the related test. The patient information was accessed only by the operator via tablets. In most cases, the exercises included an initial phase of exposure to the topic through the robot and a series of neuropsychological tests whose possible answers were displayed on the tablet. The robot acquired the answers (right or wrong), response time, time of execution of the entire exercise, and number of attempts (if the exercise provided it). The evaluation percentage was calculated based on this information.

**Figure 2:**
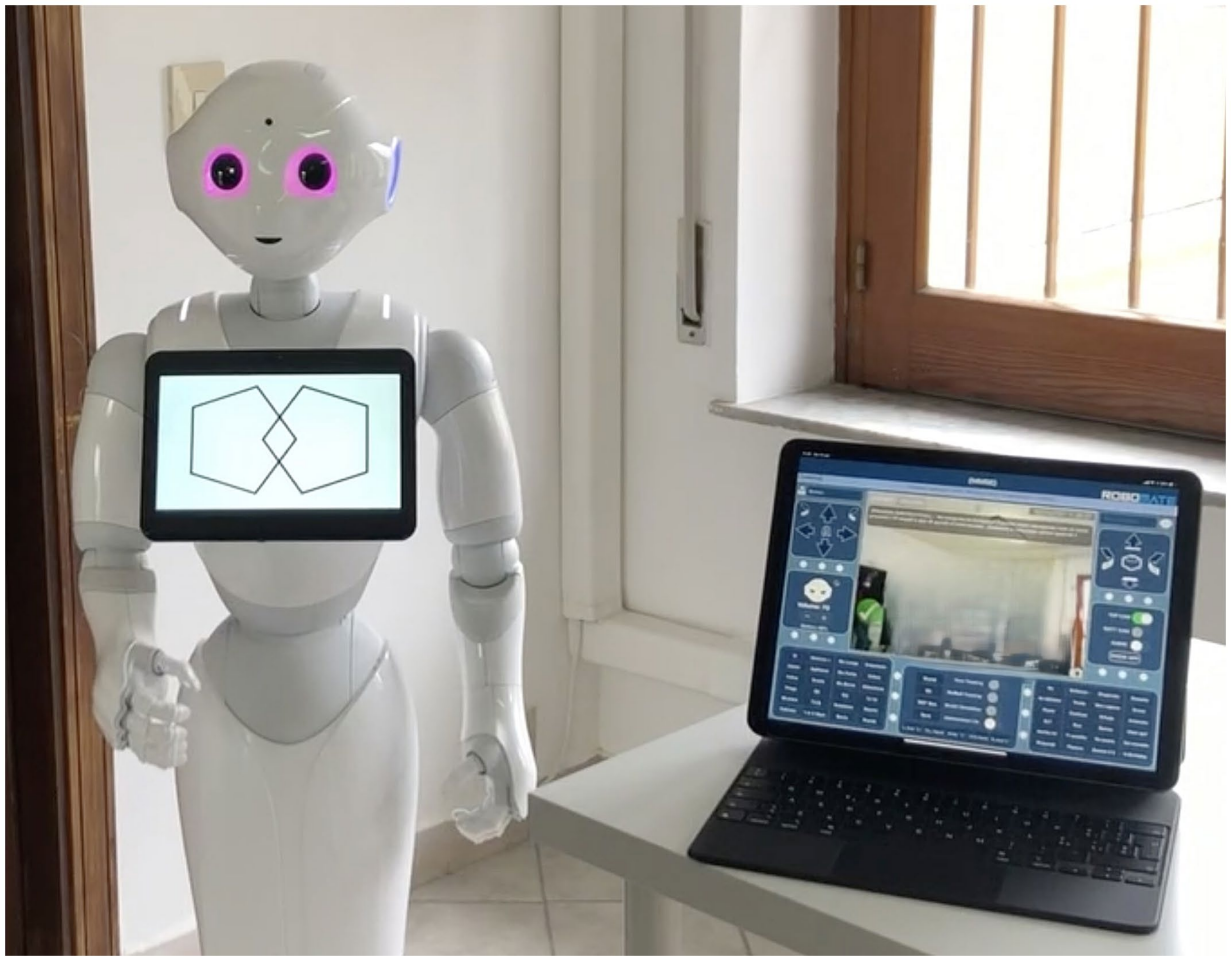
Mini Mental State Examination (MMSE) subtest “constructive praxis.”

**Figure 3:**
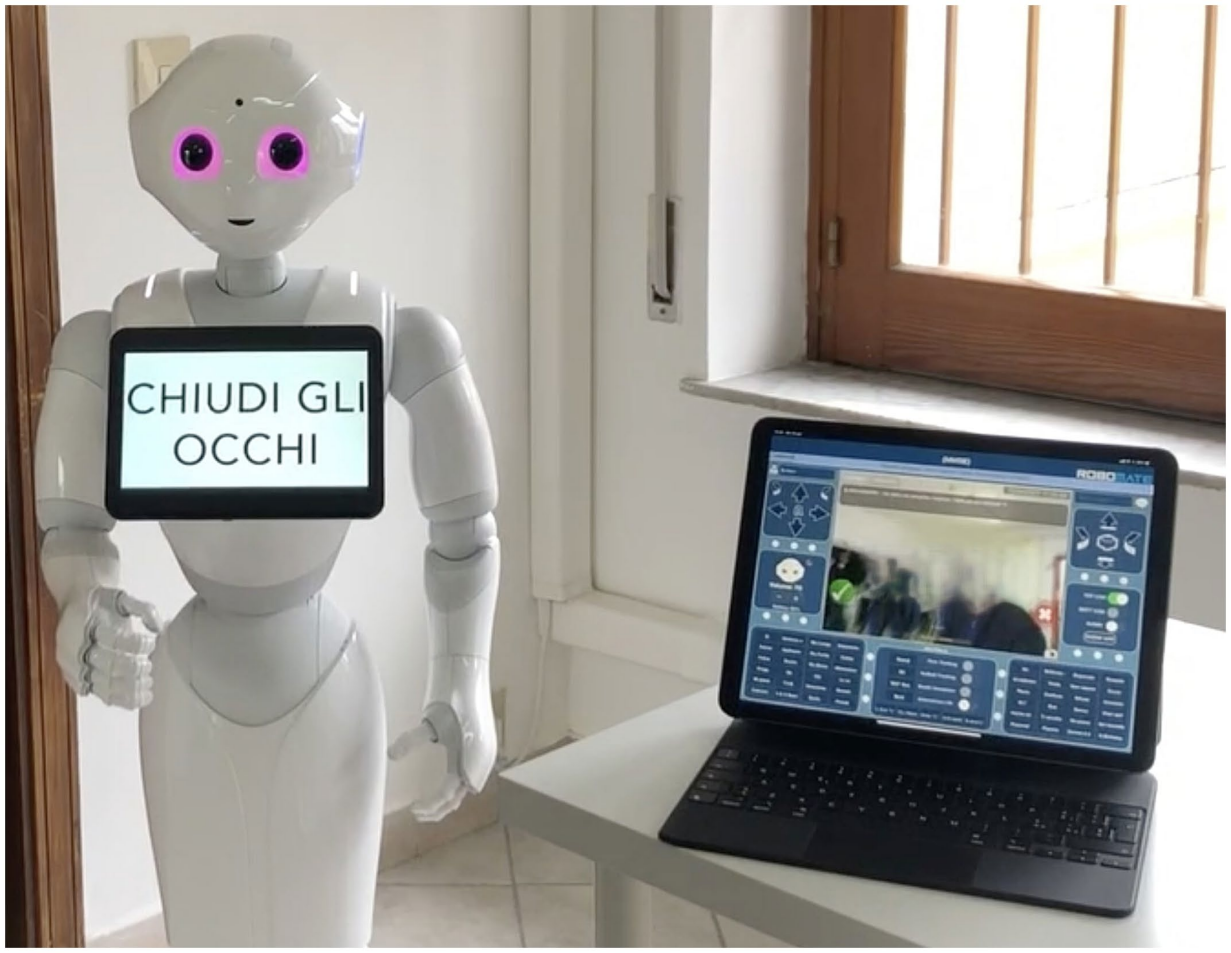
Mini Mental State Examination (MMSE) subtest “language.”

The Mini-Mental State Examination (MMSE) is the commonly used assessment of the mental state of the elderly. The cognitive areas indicated are spatial and temporal orientation, attention, memory, denomination, language, and the execution of verbal commands (Folstein et al., 1975; Figures 2, 3). We integrated this test in the robot into an administration that was normally executed by the psychologist face-to-face with the patient.

### 2.3 Procedures

One hundred patients were randomized into two groups. The patients assigned to the EG underwent an MMSE evaluation using the robot. A psychologist introduced the purpose of the study and evaluated if the patients ensured the inclusion criteria to participate to the study. Using its synthesized voice, the robot proposed the test’s questions to the patient. During the execution of the MMSE with the robot, the psychologist, had a separate tablet to monitor and record the patient’s responses. The presence of a human operator during the robot-led session was intentionally limited to a monitoring role, ensuring patient safety and intervening only in the rare case of technical issues or clinical need. The interaction, instructions, and test execution were fully managed by the robot itself. At the end of the MMSE administration, all participants completed a newly developed Satisfaction Questionnaire (SQ) designed to assess their emotional and experiential response to the testing procedure, whether administered by a human or a robot. The instrument consists of 7 items rated on dychotomic responses (yes and no) (1 = yes, 0 = no) and explores multiple dimensions, including perceived comfort, anxiety, judgment, frustration, motivation, enjoyment, and overall willingness to repeat the test. The questionnaire was created specifically for this study, drawing on existing literature concerning user experience and affective responses in human–robot interaction (Desideri et al., 2019; Holthöwer and Van Doorn, 2023; Heerink et al., 2010). The final score ranges from 0 to 7 (if patients said yes to all questions), with lower values indicating greater overall acceptability and a more positive emotional experience.

Patients assigned to the CG were assessed by a psychologist using the standard MMSE. The patients were submitted to the same questionnaire (SQ) as the EG to investigate the acceptance, the emotional sphere and usability of the test administration by the psychologist. The cognitive test and SQ administration session lasted 30 min.

### 2.4 Statistical analysis

Descriptive statistics were computed to summarize the sociodemographic and clinical characteristics of the sample. Continuous variables were expressed as means and standard deviations, while categorical variables were presented as frequencies and percentages. The assumption of normality for continuous variables was assessed using the Shapiro–Wilk test. Group comparisons were conducted using the independent-samples Student’s t-test for normally distributed variables and the Mann–Whitney U test for non-normally distributed variables. Differences in categorical variables were evaluated using Chi-square (*χ*^2^) test. Spearman’s rank correlation coefficient was employed to explore potential associations between the Total SQ score and MMSE performance within each group.

All analyses were performed using the open-source R4.2.2 software package provided by the R Foundation for Statistical Computing, Vienna, Austria. A two-tailed significance level of 95% was set, with an alpha error of 5%. Statistical significance was defined as a *p*-value of less than 0.05.

## 3 Results

The sociodemographic characteristics of the two groups are shown in Table 1. The intergroup analysis (EG vs. CG) revealed significant differences in age (*p* = 0.003) and Total SQ (*p* = 0.01), while no significant differences were observed in years of education (*p* = 0.42) and MMSE (*p* = 0.18). As shown in Table 2, the Chi-square test highlighted significant differences between groups in several items of the SQ: SQ2 (*χ*^2^ = 9.76; *df* = 1; *p* = 0.002), SQ4 (*χ*^2^ = 5.02; *df* = 1; *p* = 0.02), SQ5 (*χ*^2^ = 25.35; *df* = 1; *p* < 0.001), SQ6 (*χ*^2^ = 7.68; *df* = 1; *p* = 0.006) and SQ7 (*χ*^2^ = 7.56; *df* = 1; *p* = 0.006). No significant associations were found between Total SQ scores and MMSE performance within either group.

**Table 1:** Socio-demographic and characteristics of groups.

Variables	EG	CG	*p*
N. subject	50	50	
Male	25 (50%)	21 (42%)	
Female	25 (50%)	29 (58%)	
Age	72.68 ± 7.63	76.78 ± 5.67	0.003
Education	8 (8–13)	8 (5.5–13)	0.42
Total AQ	3 (3–3.75)	4 (3–4)	0.01
MMSE	22.35 (17.47–25.17)	19.9 (16.54–24)	0.18

Data are presented as means ± standard deviations for normally distributed variables and medians (first and third quartiles) for non-normally distributed variables.

**Table 2:** Comparison of responses (SQ1–SQ7) between EG and CG groups.

Questionnaire items	Responses	EG	CG	*χ* ^2^	*p*
I felt uncomfortable (SQ1)	No	40 (80%)	32 (64%)	2.43	0.12
	Yes	10 (20%)	18 (36%)		
I felt judged (SQ2)	No	40 (80%)	24 (48%)	9.76	0.002
	Yes	10 (20%)	26 (52%)		
I felt motivated to complete the test (SQ3)	No	5 (10%)	13 (26%)	3.32	0.07
	Yes	45 (90%)	37 (74%)		
I had the desire to stop the test (SQ4)	No	47 (94%)	38 (76%)	5.02	0.02
	Yes	3 (6%)	12 (24%)		
I felt frustrated (SQ5)	No	45 (90%)	20 (40%)	25.32	<0.001
	Yes	5 (10%)	30 (60%)		
I enjoyed answering the questions (SQ6)	No	6 (12%)	19 (38%)	7.68	0.006
	Yes	44 (88%)	31 (61%)		
I would gladly repeat the test at the next visit (SQ7)	No	4 (8%)	16 (32%)	7.56	0.006
	Yes	46 (92%)	34 (68%)		

EG, Experimental Group; CG, Control Group; SQ, Satisfaction Questionnaire.

## 4 Discussion

**Figure 4:**
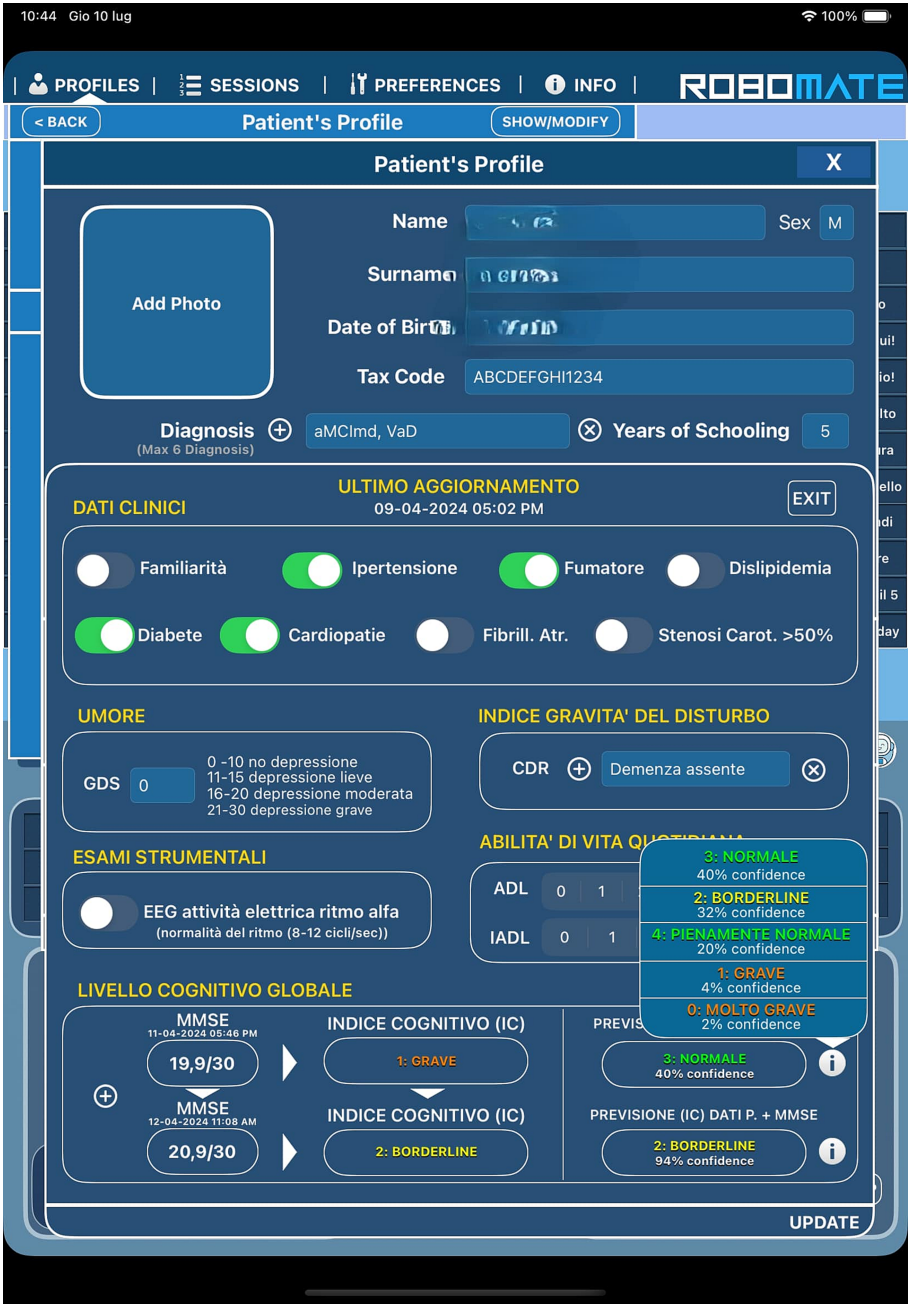
Patient’s profile with clinical data collection and results of predictive model with Mini Mental State Examination (MMSE) scores.

This study examines the feasibility of employing an assistive humanoid robot in cognitive testing. We used Pepper to administer the MMSE to the MCI population to detect the global cognitive level. Furthermore, we investigated the eventual bias in the MMSE score based on the modality of administration (robot or healthcare professional). The quality of the interaction between humans and robots was investigated using a self-report questionnaire with seven items that investigated the emotional response of the population during the interaction and the level of frustration. Our results suggest that there were significant differences about the level of frustration and perceived discomfort as highlighted from the satisfaction questionnaire administered at the end of the performance Furthermore, 80% of the EG declared that they did not feel judged during the evaluation compared to the CG (48%), in which the test was administered by a psychologist (Table 2). Another important result that emerged from the study was a decrease in the level of frustration during performance. Specifically, 90% of the EG did not report frustration during performance compared to 40% of the CG. This finding suggests that the perception of cognitive performance by a robot rather than a human may significantly influence a patient’s emotional experience. This reduction in frustration could result from the impersonal and judgment-free nature of the robot, which avoids the emotional and relational dynamics that may arise during an interaction with a human (Laban and Cross, 2023). Previous studies (Rossi et al., 2018) highlighted the potential of employing humanoid robots for the administration of cognitive tests. In addition, we did not observe significant differences between the MMSE scores obtained by the EG and CG (Table 1), suggesting that cognitive performance was neither impaired nor enhanced by the presence of the robot. These findings are in line with previous studies showing that humanoid robots can support cognitive screening with comparable diagnostic reliability to standard tools such as the MMSE (Tuncer et al., 2023). Several advantages associated with the robot may help explain the more positive ratings it received in certain instances. These include its non-judgmental appearance, predictable and standardized interaction style, and the potential novelty or curiosity effect, which may reduce performance-related stress and increase user engagement during cognitive assessment (Holthöwer and Van Doorn, 2023). The software integrated into the robot was supported by an artificial intelligence system with the advantage of providing the correct test score in real time. Furthermore, it is possible to track the long-term test performance immediately with easy data retrieval and trend processing. The system was provided with an MLM that allowed the processing and matching of scores obtained from clinical data, and using algorithm processing, allowed cognitive decline to be predicted over time. According to a previous study starting from the training of the predictive model was performed with the construction of a defined dataset, it was possible to make predictions on the evolution of these data (Figure 4). The system has a predictive function linked to the evolution of a patient’s clinical state, which is obtained by matching clinical data with neuropsychological test scores. The results obtained were significant, with a diagnostic accuracy of 86%, sensitivity of 72%, and specificity reached a value of 91% (Formica et al., 2023). These results are encouraging because the level of accuracy is quite high and indicate the high reliability of the predictive model. Considering the variability of neurodegenerative diseases, it is essential to collect an ever-increasing amount of data to develop more accurate and reliable predictive models. Data collection could be encouraged by the fact that patients seem to appreciate Pepper, attributing social abilities to it, which benefited the overall interaction with the robot, fostering wider data collection. The use of AI-driven robotic technology for assessment and collection of medical history data has made it possible to monitor patients’ clinical conditions over time; providing the possibility to robot programming session for cognitive rehabilitation in MCI patients (Demetriadis et al., 2015), robotic rehabilitation systems contribute not only to motor recovery but also to the creation of a socially engaging and supportive environment. This social dimension plays a key role in increasing patient acceptance of the technology, thereby improving both the feasibility and the usability of the therapeutic intervention. In fact, our results demonstrated good level of acceptance resulting from the scores of the SQ (see Table 2). Generally, the population examined seems to have a good future prospective to use Pepper for the assessment of performances favoring social interaction. The overall positive feedback from patients in the robot condition suggests that such interactions were well tolerated and may have elicited a more emotionally positive experience during cognitive testing. This could be related to factors such as the robot’s non-judgmental, which might have contributed to reducing anxiety during the task. Social robots can elicit social interactions that closely resemble human-to-human exchanges. Their use in cognitive testing may enhance the ecological validity of the assessment process, while simultaneously enabling automated tracking of gaze behavior. Moreover, unlike human examiners, social robots offer the advantage of minimizing distracting facial cues—such as spontaneous gaze shifts—thereby ensuring a more standardized and replicable evaluation of visual attention (Desideri et al., 2019). One study showed how education level can influence the bias to interact with a robot rather than a human being (Szczepanowski et al., 2020). For our sample size, we attempted to obtain two homogeneous groups according to age and education (Table 1) to exclude this bias. However, these findings may change with a larger sample size, thereby reducing or increasing this bias. While earlier studies on social robots have focused on feasibility or emotional perception, our work compared robot- and human-led administration of a widely used cognitive screening tool (MMSE), under ecologically valid clinical conditions in patients with MCI. Additionally, the SQ assessed emotional and motivational components of patient experience. A further distinctive element is the integration of AI system-based software into the robotic system. The robot was directly integrated with the software that provided real-time scoring, longitudinal performance tracking, and predictive modeling of cognitive decline, based on neuropsychological outcomes and clinical risk factors that was registered during the collection of clinical data by the neurologist. Such a system enables early stratification of cognitive trajectories, with proven high diagnostic accuracy, sensitivity, and specificity. Finally, the positive reception of the robot by patients—potentially driven by its perceived social skills—represents an important factor in facilitating widespread data collection for future prospective. Nonetheless, limitations include the need for external validation of the acceptability tool, the relatively small sample size, and the absence of baseline data on participants’ technological familiarity, which may affect interaction outcomes. Finally, the sample size, while adequate for preliminary comparison, may not capture subtler effects or broader generalizability.

## 5 Conclusion

These results revealed interesting questions about the psychological implications of human-robot interactions. This dynamic appears to create a more relaxed environment, enabling patients to perform assessments accurately without the anxiety associated with human judgment. Such a shift could have significant implications for the quality of cognitive testing and diagnoses as well as for the overall patient experience, reducing the risk of errors linked to psychological stress. In the future, the integration of intelligent machines to manage neurodegenerative diseases may be important. Advanced algorithms could allow for more accurate predictions of disease progression based on real-time data collection and analysis. This approach enables the monitoring of a patient’s clinical condition and the definition of the most effective, personalized, and early therapeutic treatment. Therefore, the adoption of robots and advanced technologies can enhance diagnostic reliability as well as transform the entire therapeutic landscape, making the management of neurodegenerative diseases more precise and targeted for early interventions.

## Data Availability

The datasets presented in this article are not readily available because the dataset are available upon request from the corresponding author. Requests to access the datasets should be directed to desiree.latella@irccsme.it.

## Footnotes

^1^ https://www.random.org

^2^ https://japansociety.org/news/meet-pepper-the-worlds-first-humanoid-robot-that-reads-human-emotions/

## References

[ref1] Ataollahi Eshkoor S. Mun C. Y. Ng C. K. Hamid T. A. (2015). Mild cognitive impairment and its management in older people. Clin. Interv. Aging, 10, 687–693. doi: 10.2147/CIA.S73922, PMID: 25914527 PMC4401355

[ref2] Berres M. Monsch A. U. Spiegel R. (2021). Using historical data to facilitate clinical prevention trials in Alzheimer disease? An analysis of longitudinal MCI (mild cognitive impairment) data sets. Alzheimer's Res Ther 13:97. doi: 10.1186/s13195-021-00832-5, PMID: 33962665 PMC8106156

[ref3] Chua S. I. L. Tan N. C. Wong W. T. Allen J. C. Jr. Quah J. H. M. Malhotra R.. (2019). Virtual reality for screening of cognitive function in older persons: comparative study. J. Med. Internet Res. 21:e14821. doi: 10.2196/14821, PMID: 31373274 PMC6694729

[ref4] Coronado E. Kiyokawa T. Ricardez G. A. G. Ramirez-Alpizar I. G. Venture G. Yamanobe N. (2022). Evaluating quality in human-robot interaction: a systematic search and classification of performance and human-centered factors, measures and metrics towards an industry 5.0. J. Manuf. Syst. 63, 392–410. doi: 10.1016/j.jmsy.2022.04.007

[ref5] Demetriadis S. Giannouli V. Sapounidis T. (2015). “Robot programming and tangible interfaces for cognitive training” in Handbook of research on innovations in the diagnosis and treatment of dementia. eds. Bamidis P. Tarnanas I. Hadjileontiadis L. Tsolaki M. (Hershey, PA: IGI Global Scientific Publishing), 196–223.

[ref6] Desideri L. Ottaviani C. Malavasi M. Di Marzio R. Bonifacci P. (2019). Emotional processes in human-robot interaction during brief cognitive testing. Comput. Hum. Behav. 90, 331–342. doi: 10.1016/j.chb.2018.08.013

[ref7] Fania A. Monaco A. Amoroso N. Bellantuono L. Cazzolla Gatti R. Firza N.. (2023). A dementia mortality rates dataset in Italy (2012–2019). Sci. Data 10:564. doi: 10.1038/s41597-023-02461-z, PMID: 37626087 PMC10457292

[ref8] Folstein M. F. Folstein S. E. McHugh P. R. (1975). “Mini-mental state”: a practical method for grading the cognitive state of patients for the clinician. J. Psychiatr. Res. 12, 189–198. doi: 10.1016/0022-3956(75)90026-6, PMID: 1202204

[ref9] Formica C. Bonanno L. Giambò F. M. Maresca G. Latella D. Marra A.. (2023). Paving the way for predicting the progression of cognitive decline: the potential role of machine learning algorithms in the clinical management of neurodegenerative disorders. J. Pers. Med. 13:1386. doi: 10.3390/jpm13091386, PMID: 37763152 PMC10533011

[ref10] Formica C. Bonanno M. Sorbera C. Quartarone A. Giambò F. M. Marra A.. (2024). Smartphone-based cognitive telerehabilitation: a usability and feasibility study focusing on mild cognitive impairment. Sensors 24:525. doi: 10.3390/s24020525, PMID: 38257618 PMC10820398

[ref11] Gervasi G. Bellomo G. Mayer F. Zaccaria V. Bacigalupo I. Lacorte E.. (2020). Integrated care pathways on dementia in Italy: a survey testing the compliance with a national guidance. Neurol. Sci. 41, 917–924. doi: 10.1007/s10072-019-04184-9, PMID: 31836948 PMC7160089

[ref12] Giannouli V. (2023). Financial capacity assessments and AI: a Greek drama for geriatric psychiatry? Int. J. Geriatr. Psychiatry 38:e6008. doi: 10.1002/gps.6008, PMID: 37724603

[ref13] Giannouli V. Kampakis S. (2024). Can machine learning assist us in the classification of older patients suffering from dementia based on classic neuropsychological tests and a new financial capacity test performance? J. Neuropsychol. 1–14. doi: 10.1111/jnp.12409 PMC12424109 39696757

[ref14] Graham S. A. Lee E. E. Jeste D. V. Van Patten R. Twamley E. W. Nebeker C.. (2020). Artificial intelligence approaches to predicting and detecting cognitive decline in older adults: a conceptual review. Psychiatry Res. 284:112732. doi: 10.1016/j.psychres.2019.112732, PMID: 31978628 PMC7081667

[ref15] Heerink M. Kröse B. Evers V. Wielinga B. (2010). Assessing acceptance of assistive social agent technology by older adults: the almere model. Almere, The Netherlands. Int. J. Soc. Robot. 2, 361–375. doi: 10.1007/s12369-010-0068-5

[ref16] Holthöwer J. Van Doorn J. (2023). Robots do not judge: service robots can alleviate embarrassment in service encounters. J. Acad. Mark. Sci. 51, 767–784. doi: 10.1007/s11747-022-00862-x, PMID: 35463183 PMC9019535

[ref17] Katsanos A. H. Lee S. F. Cukierman-Yaffe T. Sherlock L. Muniz-Terrera G. Canavan M.. (2023). World-wide variations in tests of cognition and activities of daily living in participants of six international randomized controlled trials. Cereb. Circ. Cogn. Behav. 5:100176. doi: 10.1016/j.cccb.2023.100176, PMID: 37501909 PMC10368824

[ref18] Koo B. M. Vizer L. M. (2019). Mobile technology for cognitive assessment of older adults: a scoping review. Innov. Aging 3:igy038. doi: 10.1093/geroni/igy038, PMID: 30619948 PMC6312550

[ref19] Laban G. Cross E. S. (2023). Sharing with robots: why do we do it and how does it make us feel? PsyArxiv. doi: 10.31234/osf.io/2azpq

[ref20] Lopez O. L. (2013). Mild cognitive impairment. Continuum Lifelong Learn. Neurol. 19, 411–424. doi: 10.1212/01.CON.0000429175.29601.97, PMID: 23558486 PMC3915547

[ref21] Morris J. C. Storandt M. Miller J. P. McKeel D. W. Price J. L. Rubin E. H.. (2001). Mild cognitive impairment represents early-stage Alzheimer disease. Arch. Neurol. 58, 397–405. doi: 10.1001/archneur.58.3.397 11255443

[ref22] Rossi S. Santangelo G. Staffa M. Varrasi S. Conti D. di Nuovo A. (2018). Psychometric evaluation supported by a social robot: personality factors and technology acceptance. In Proceedings of the 2018 27th IEEE international symposium on robot and human interactive communication (RO-MAN), Nanjing, China; pp. 802–807.

[ref23] Sorrentino A. Mancioppi G. Coviello L. Cavallo F. Fiorini L. (2021). Feasibility study on the role of personality, emotion, and engagement in socially assistive robotics: a cognitive assessment scenario. Informatics 8:23. doi: 10.3390/informatics8020023

[ref24] Szczepanowski R. Cicho’n E. Arent K. Sobecki J. Styrkowiec P. Florkowski M.. (2020). Education biases perception of social robots. Eur. Rev. Appl. Psychol. 70:100521. doi: 10.1016/j.erap.2020.100521

[ref25] Takaeda K. Kamimura T. Inoue T. Nishiura Y. (2019). Reliability and acceptability of using a social robot to carry out cognitive tests for community-dwelling older adults. Geriatr Gerontol Int 19, 552–556. doi: 10.1111/ggi.13655, PMID: 30884153

[ref26] Tarnanas I. Schlee W. Tsolaki M. Müri R. Mosimann U. Nef T. (2013). Ecological validity of virtual reality daily living activities screening for early dementia: longitudinal study. JMIR Serious Games 1:e1. doi: 10.2196/games.2778, PMID: 25658491 PMC4307822

[ref27] Tuncer S. Coninx A. Demeure V. Devillers L. (2023). Human or robot? An exploratory study of the impact of robot-assisted cognitive tests on older adults' emotions. Format. Exp. HRI 1:e42792. doi: 10.58079/ra4m

[ref28] Varrasi S. Di Nuovo S. Conti D. Di Nuovo A. (2018). A social robot for cognitive assessment. In Companion of the 2018 ACM/IEEE international conference on human-robot interaction (pp. 269–270

[ref29] WHO (2020). World health statistics 2020: monitoring health for the sdgs, sustainable development goals. Available online at: https://www.who.int/publications/i/item/9789240005105 (accessed July 10, 2025).

[ref30] Yang C. Moore A. Mpofu E. Dorstyn D. Li Q. Yin C. (2020). Effectiveness of combined cognitive and physical interventions to enhance functioning in older adults with mild cognitive impairment: a systematic review of randomized controlled trials. The Gerontologist 60, e633–e642. doi: 10.1093/geront/gnz149, PMID: 31697831

